# 病毒性肺炎的流行对肺癌患者的影响和思考

**DOI:** 10.3779/j.issn.1009-3419.2020.102.13

**Published:** 2020-04-20

**Authors:** 艳红 姚, 明 路, 燕娥 刘, 宝山 曹

**Affiliations:** 1 100191 北京，北京大学第三医院肿瘤化疗与放射病科 Department of Medical Oncology and Radiation Sickness Peking University Third Hospital, Beijing 100191, China; 2 100191 北京，北京大学第三医院呼吸与危重症医学科 Department of Respiratory and Critical Care Medicine, Peking University Third Hospital, Beijing 100191, China

**Keywords:** 肺肿瘤, 病毒性肺炎, 个体化管理, Lung neoplasms, Viral pneumonia, Individualized management

## Abstract

21世纪以来，全球爆发了三次冠状病毒感染、一次流感病毒感染，均引起呼吸系统病变，严重威胁人类健康。肺癌患者多为高龄，由于肿瘤本身及经过抗肿瘤治疗后免疫功能下降，更容易感染病毒且预后更差。肺癌及其治疗后继发的疾病亦可引起患者发热及呼吸道症状，临床上需要做好与病毒性肺炎的鉴别；此外，病毒性肺炎的防控措施可能会影响肺癌患者的常规诊疗。因此，病毒性肺炎防控期间，对肺癌患者进行科学防护和个体化管理尤为重要。本文系统性总结了病毒性肺炎的流行病学及临床特征、病毒性肺炎对肺癌患者的影响、肺癌及治疗相关病变与病毒性肺炎的鉴别诊断，旨在为肺癌患者在病毒性肺炎防控期间的个体化管理提供一定指导，从而最大程度上保护患者的利益。

## Abstract (en)

The worldwide epidemic of three coronaviruses and one influenza virus in 21^st^ century have seriously threatened human health. Infection with these viruses can cause respiratory symptoms. The patients with lung cancer are more susceptible to viral infection and have a worse prognosis due to the advanced age and the systemic immunosuppressive state caused by malignancy itself and the anticancer treatments. In addition, without sufficient clinical awareness, a missed diagnosis of viral pneumonia may occur due to the fever and respiratory symptoms caused by lung cancer and its secondary diseases. Furthermore, control measures against viral outbreaks may interfere with routine diagnosis and treatment of lung cancer patients. Therefore, scientific protection and individualized management of lung cancer patients are particularly important during virus epidemic prevention and control. Here, we systematically reviewed the epidemiological and clinical characteristics of viral pneumonia, its impact on patients with lung cancer and the differential diagnosis of lung cancer-related respiratory manifestations, aiming to provide guidance for the individual management of lung cancer patients during the prevention and control of viral pneumonia epidemic.

从2003年严重急性呼吸综合征（severe acute respiratory syndrome, SARS）的冠状病毒流行^[1]^，到近几年来每年冬春季的流感病毒的流行^[2, 3]^，再到2019年底的新型冠状病毒（2019 novel coronavirus, 2019-nCoV）的爆发^[4, 5]^，人们切实体会到呼吸道病毒对人类健康存在严重的危害性。除了冠状病毒之外，其他常见的可导致肺炎的病毒还包括流行性感冒病毒、腺病毒、副流感病毒、鼻病毒及呼吸道合胞病毒等^[6-9]^。人群对上述病毒普遍易感，有心血管疾病、糖尿病、慢性肺病、肿瘤等基础疾病的患者更是病毒性肺炎的高危易感人群^[8, 9]^。

全球肺癌的发生率和死亡率均占肿瘤第一位^[10]^。因肿瘤本身或经过手术、化疗、放疗等治疗后，会出现免疫功能下降，且大部分肺癌患者是伴有基础疾病的高龄患者，因此更容易发生病毒感染^[11-13]^，而且发展为重症及危重症肺炎的比例更高，预后更差^[14]^。门诊预检分诊、线上预约和诊疗、院内感染防控等措施减少了肺癌患者的感染机会，但疫情期间，重大公共卫生事件响应级别可能会影响肺癌患者的常规诊疗，特别是初诊患者和复发或进展的患者。因此，在病毒性肺炎防控期间，对于肺癌患者，科学防护和个体化管理是医院及医生亟待解决的重要问题。

目前，国内外关于病毒性肺炎对肺癌患者影响的相关研究较少，疫情期间如何优化肺癌患者诊疗策略的研究和指南缺乏。本文系统性回顾了病毒性肺炎的流行病学及临床特征、病毒性肺炎对肺癌患者的影响、病毒性肺炎与肺癌本身及治疗后继发性疾病的鉴别要点，旨在为肺癌患者在疫情期间的个体化管理提供一定的指导。

## 1 病毒性肺炎的流行病学及临床特征

几项多中心研究结果显示，我国成人社区获得性肺炎患者中病毒检出率为15.0%-34.9%^[8]^，除了近期爆发流行的新型冠状病毒之外，其他常见的病毒还包括流行性感冒病毒、腺病毒、副流感病毒、鼻病毒、呼吸道合胞病毒、人博卡病毒和人偏肺病毒等^[8]^。我国的流行病学调查研究^[9]^结果显示：各年龄段人群对上述呼吸道病毒普遍易感。其中呼吸道合胞病毒、副流感病毒、人博卡病毒、人偏肺病毒、腺病毒在儿童多见，而流感病毒是成年人和老年人急性下呼吸道病毒感染的主要病原体。对于肺癌患者，最值得关注的是流感病毒和此次湖北武汉地区爆发流行的新型冠状病毒。

流行性感冒（简称流感）是一种常见的呼吸道传染病，具有突然爆发、迅速扩散、易传染的特点，每年流行且有季节性，冬春季高发^[7]^。流感的发病率高，人群普遍易感。流感病毒通常导致上呼吸道感染，并非所有感染者都会出现肺炎。常见的流感样症状包括急性起病的发热、畏寒、乏力、头痛、全身酸痛等。如果在此基础上出现干咳、呼吸困难、反复发热、心动过速、低氧血症、发绀、肺部啰音；血常规白细胞计数不升高或减低；胸部影像学检查见新发浸润影；对抗菌药物治疗反应慢或无反应，则可以做出流感病毒肺炎的初步判断^[9]^。呼吸道标本中检测出流感病毒核酸或抗原有助于确诊^[8]^，治疗药物主要为奥司他韦、扎那米韦等^[8]^。

新型冠状病毒肺炎（2019 novel coronavirus disease, COVID-19）于2019年底在中国武汉爆发^[4, 5]^，继而疫情蔓延至全国。COVID-19由2019-nCoV引起，该病毒被命名为“SARS-CoV-2”（severe acute respiratory syndrome coronavirus 2），属于冠状病毒科的β属^[15]^。目前所见传染源主要是新型冠状病毒感染的患者。无症状感染者也可能成为传染源。经呼吸道飞沫和接触传播是主要的传播途径。人群普遍易感。COVID-19潜伏期为1 d-14 d，多为3 d-7 d^[15]^。主要表现为发热、干咳、呼吸困难，亦可表现为肌痛、头痛、咽痛、胸痛、腹泻、恶心、呕吐等^[5]^。化验结果表现为外周血白细胞减少，淋巴细胞减少，丙氨酸转氨酶、天冬氨酸氨基转移酶、肌酸肌酶、C反应蛋白升高。典型的胸部影像学表现为双肺单发或多发斑片状磨玻璃影，以肺外周胸膜下分布为著，严重者可进展至双肺弥漫实变。呼吸道标本中检测出新型冠状病毒核酸或血清新型冠状病毒特异性抗体阳性有助于确诊，但存在一定的假阴性率。粗病死率为2.3%，多为老年人和有基础病的患者^[5]^。目前缺乏循证医学证实的有效治疗药物^[15]^。

## 2 病毒性肺炎对肺癌患者的影响

### 2.1 病毒性肺炎对肺癌患者的直接影响

病毒性肺炎严重威胁着肿瘤患者的预后，特别是肺癌患者。在呼吸道病毒季节性流行期间，肿瘤患者发生病毒感染后，约20%可发展为病毒性肺炎^[16]^。Kunisaki等^[17]^报道肿瘤患者流感病毒的感染率为21%-33%，死亡率为11%-33%。墨西哥和阿根廷的研究结果^[18, 19]^显示，2009年H1N1流感防控期间住院肿瘤患者的病毒性肺炎感染率为43%-66%，感染后30 d内死亡率为12.7%-18.5%。SARS期间，德国学者发现肿瘤患者感染后死亡率高达20.2%（2/9）^[20]^。

近期COVID-19期间，梁文华等^[13]^的调查结果显示，1, 590例病史完整的中国COVID-19患者中18例为肿瘤患者，其中肺癌最常见（5/18, 28%）；与无肿瘤病史患者（8%）相比，肿瘤患者（39%）具有更高的严重事件风险（*P*=0.000, 3）^[13]^。钟南山等^[21]^分析了中国1, 099例确诊COVID-19的患者，其中10例为肿瘤患者。肿瘤患者重症比例（30%）显著高于非肿瘤患者（16%）。武汉中南医院分析了138例COVID-19患者，其中10例为肿瘤患者，入住ICU的比例（40%）明显高于非肿瘤人群（25%）^[22]^。中国疾病预防控制中心近期报道了截止到2020年2月11日20, 982例病史明确的COVID-19患者中，107例为肿瘤患者，粗死亡率为5.6%，显著高于整体人群的粗病死率（2.3%）^[5]^。这些肿瘤患者的预后还与年龄因素有关，梁文华等^[13]^的调查研究中肿瘤患者的中位年龄（63.1岁）显著高于非肿瘤患者（48.7岁）。钟南山等^[21]^报道的研究中，年龄越大，重症比例越高。肿瘤患者的重症比例（30%）与65岁以上人群的重症比例（29%）类似^[21]^。近期病理检查发现，COVID-19患者的肺部损伤严重，表现为水肿、蛋白渗出、肺泡壁细胞反应性增生，伴散在炎性细胞、多核巨细胞浸润^[23]^。综上可见，肿瘤患者特别是肺癌患者感染COVID-19后，发展为重症和死亡的风险可能更高。尽管目前因缺乏肿瘤患者风险暴露的相关数据，尚不能得出肿瘤患者更容易发生COVID-19的结论，但对指导肿瘤患者进行科学防护尤为重要。

### 2.2 病毒性肺炎对肺癌患者的间接影响

病毒流行对肿瘤患者心理和诊疗势必也会产生很大影响。2003年SARS冠状病毒流行期间，多项研究显示肺癌患者的诊治均受到不同程度的影响。台湾荣名总医院调查^[24]^发现，63.8%的肺癌患者害怕住院，36.2%的患者认为SARS比肺癌更严重、更致命，4%的患者因担心感染而拒绝进一步化疗，化疗延迟发生率为3%^[24]^。香港威尔斯亲王医院结直肠外科中心门诊出诊医生减少52%-59%，入院人数减少51%，外科手术减少32%，结直肠的小型择期手术等候时间延长了5个月^[25]^。多伦多大学放射肿瘤学中心发现肿瘤治疗咨询量减少21%，放疗患者数量减少15%^[26]^。此外，支气管镜检查是肺癌患者确诊以及后续诊治的重要工具。SARS-CoV-2主要通过呼吸道飞沫传播、空气传播及接触传播，而支气管镜检查过程中操作相关医务人员须与患者近距离接触，患者咳嗽、用力呼吸等可产生大量飞沫或气溶胶，污染室内设备、空气、人员，甚至喷射或飞溅至操作人员的角膜、皮肤、衣物等，具有较高风险的患-医和患-患之间交叉感染。同时由于COVID-19患者潜伏期长，症状不典型，因此，在当前COVID-19疫情期间，支气管镜检查受到很大程度的限制，这必然会对肺癌患者的诊断造成很大影响。到目前为止，COVID-19较SARS影响范围更广，涉及人群更多，因此COVID-19对肿瘤患者特别是肺癌患者的心理和诊疗的不利影响可能更大，需要进一步调查研究明确影响及找寻精细化管理方案。

## 3 病毒性肺炎与肺癌及其治疗后继发疾病的鉴别诊断

肺癌患者在诊治期间可能存在发热^[27, 28]^及呼吸道相关症状，包括化疗后肺部感染、药物性肺损害（尤其是靶向药物和免疫检测点抑制剂导致的间质性肺炎）、放射性肺炎等。因此，在病毒性肺炎防控期间，临床上需要做好上述疾病与病毒性肺炎相鉴别的工作，其鉴别要点在于流行病学史、治疗史和疾病演变过程，必要时进行细菌学和病毒等病原学检测。医生需要熟悉肺癌及继发性疾病的特征及肺癌患者发热常见病因，从而更好地指导患者诊疗，以减少患者对病毒性肺炎的恐慌和降低感染风险。针对目前COVID-19，应掌握COVID-19与肺癌及治疗后继发性疾病的鉴别要点（表 1）^[21, 29-33]^。同时肺癌伴发热的患者，也需要注意COVID-19与肿瘤性发热^[27]^、感染性发热、中性粒细胞减少性发热^[28]^和药物性发热相鉴别。

**1:** COVID-19与肺癌及肺癌治疗后继发性疾病的鉴别 Difference between lung cancer, treatment-related diseases and COVID-19

Disease	History	Symptom	Laboratory data	Image study
COVID-19^[4, 5, 21]^	Epidemiological history	Fever, sore throat, muscle ache, fatigue, cough, and may be accompanied by dyspnea in severe cases	Leucocytes↓ Lymphocytes↓ SARS-CoV-2 test (+)	Single or multiple mottling or ground-glass opacity or condensation shadows in bilateral lung, mainly in the peripheral lung, and pleural suffusion is rare
Lung cancer with obstructive pneumonia^[29]^	Lung cancer history	No special symptoms, and may shows as fever, cough and shortness of breath	Leucocytes↑ Neutrophils↑ Procalcitonin↑ Bacteriology test (+)	Bronchial stenosis or occlusion in the proximal part of the lesion and pulmonary atelectasis or infiltration or condensation shadows in the distal part of the lesion
Radiation pneumonia^[30]^	Radiotherapy history, mostly happened in 1 mon-3 mon after radiation	Cough usually precedes fever, and may be accompanied by dyspnea in severe cases	No special	Mottling or ground-glass opacity or condensation shadows in the radiation field
Drug-induced interstitial pneumonia^[31, 32]^	Chemotherapy or molecular targeted therapy history	Progressive dyspnea and cough	No special	Widespread patchy or diffuse consolidation or ground-glass opacity with or without intralobular reticular opacity and septal thickening
Immune checkpoint inhibitor therapy-related pneumonitis^[33]^	Treatment history of PD-1, PD-L1 or CTLA-4	Cough and dyspnea are usual, and fever is rare	No special	Multiple or diffuse ground-glass opacity or mesh shadow or condensation shadows in lung
COVID-19: 2019 novel coronavirus disease; SARS-CoV-2: severe acute respiratory syndrome coronavirus 2; PD-1: programmed cell death protein 1; PD-L1: programmed cell death protein ligand 1; CTLA-4: cytotoxic T lymphocyte associated antigen 4.				

## 4 病毒性肺炎防控期间肺癌患者的个体化管理建议

病毒性肺炎对肺癌患者包括感染者及非感染者有直接和间接的影响，特别是COVID-19。截至2020年3月21日，COVID-19已蔓延至全球100多个国家，确诊病例超过20万人，中国、韩国、意大利、美国等多个国家采取了最高级别的防控措施，世界卫生组织已将COVID-19的风险级别提升为“非常高”。因此，病毒性肺炎特别是COVID-19防控期间，对肺癌患者进行个体化管理尤为重要。

### 4.1 COVID-19防控期间肺癌诊疗建议

首先，医护人员要掌握病毒性肺炎的流行病学和临床特征，指导患者科学防护，戴口罩，勤洗手，多通风，避免人群聚集，减少医院就诊频率和医院停留时间，确保安全距离1 m以上；饮食指导可参考《关于防治新型冠状病毒感染的饮食营养专家建议》^[34]^。其次，严格防控病毒流行期间，医护人员应制定预防交叉感染的应急预案，包括但不限于医务人员防护、门诊、病房、日间病房诊疗预案，最大程度上避免感染的发生；医护人员在行医过程中，按照各个诊疗区域的防护标准严格进行自我防护，在与不同患者接触时注意手卫生及诊疗器械消毒，预防患-医和患-患之间的交叉感染；治疗需要依据患者肿瘤程度、患者身体状况、区域管控程度、药品可及性制定个体化治疗方案。最后，对于发现疑似或感染的患者应遵循“早发现、早报告、早隔离、早治疗”原则，对于严格防控的病毒性感染的确诊病例应转至定点医院进行治疗，康复出院后应观察2个潜伏期以上，确保无复发及传染性，方可考虑后续治疗。

### 4.2 COVID-19防控期间肺癌患者的管理建议

COVID-19流行期间，对于肺癌患者的管理有如下建议：①给予科学的防护和饮食、运动指导；②完成肺癌患者所受影响程度及心理状态问卷调研，以帮助优化诊疗方案；③治疗上遵循“就近原则”，建立“主诊医师主动管理或科室主动管理”制度，充分发挥网络平台功能，指导患者诊疗及毒副反应管理；④对于初诊和可行根治性手术的患者，建议多学科综合诊疗（multi-disciplinary treatment, MDT）团队充分评估，确定诊疗方案和手术时机；对于根治性术后患者，可适当推迟辅助化疗的时间，对于年老体弱者、基础疾病多、术后合并并发症者及术后体能恢复欠佳者，可密切观察，不建议过早行辅助化疗；对于不可手术的局部进展期或晚期肺癌或进展/复发的肺癌患者：通过MDT讨论，有计划、合理地应用化疗、放疗、靶向和免疫治疗等手段。具体可参考徐燕等制定的《新型冠状病毒肺炎疫情期间肺癌患者临床管理》^[35]^。

总之，病毒性肺炎对肺癌患者危害严重，医护人员应结合病毒性肺炎流行特点进行防护指导，依据患者肿瘤情况、身体情况、肿瘤治疗的获益风险比、防控级别等制定个体化诊疗方案。开展多中心研究探讨COVID-19对肺癌患者心理、诊疗和预后的影响及原因，有助于未来更好地帮助肺癌患者应对此类公共卫生事件，从而最大程度的保护肺癌患者的身心健康。
